# An In Silico Analysis of Synthetic and Natural Compounds as Inhibitors of Nitrous Oxide Reductase (N_2_OR) and Nitrite Reductase (NIR)

**DOI:** 10.3390/toxics11080660

**Published:** 2023-08-01

**Authors:** Radhakrishnan Narayanaswamy, Vasantha-Srinivasan Prabhakaran, Mysoon M. Al-Ansari, Latifah A. Al-Humaid, Pragya Tiwari

**Affiliations:** 1 Department of Biochemistry, Saveetha Medical College and Hospital, Saveetha Institute of Medical and Technical Sciences, Chennai 602105, Tamil Nadu, India; 2 Department of Bioinformatics, Saveetha School of Engineering, Saveetha Institute of Medical and Technical Sciences, Chennai 602105, Tamil Nadu, India; vasanth.bmg@gmail.com; 3 Department of Botany and Microbiology, College of Science, King Saud University, Riyadh 11451, Saudi Arabia; myalansari@ksu.edu.sa (M.M.A.-A.); alhumaid@ksu.edu.sa (L.A.A.-H.); 4 Department of Biotechnology, Yeungnam University, Gyeongsan-si 38541, Republic of Korea

**Keywords:** nitrification, denitrification, nitrous oxide reductase (N_2_OR), nitrite reductase (NIR), *Paracoccus denitrificans*, *Hyphomicrobium denitrificans*

## Abstract

Nitrification inhibitors are recognized as a key approach that decreases the denitrification process to inhibit the loss of nitrogen to the atmosphere in the form of N_2_O. Targeting denitrification microbes directly could be one of the mitigation approaches. However, minimal attempts have been devoted towards the development of denitrification inhibitors. In this study, we aimed to investigate the molecular docking behavior of the nitrous oxide reductase (N_2_OR) and nitrite reductase (NIR) involved in the microbial denitrification pathway. Specifically, in silico screening was performed to detect the inhibitors of nitrous oxide reductase (N_2_OR) and nitrite reductase (NIR) using the PatchDock tool. Additionally, a toxicity analysis based on insecticide-likeness, Bee-Tox screening, and a STITCH analysis were performed using the SwissADME, Bee-Tox, and pkCSM free online servers, respectively. Among the twenty-two compounds tested, nine ligands were predicted to comply well with the TICE rule. Furthermore, the Bee-Tox screening revealed that none of the selected 22 ligands exhibited toxicity on honey bees. The STITCH analysis showed that two ligands, namely procyanidin B2 and thiocyanate, have interactions with both the *Paracoccus denitrificans* and *Hyphomicrobium denitrificans* microbial proteins. The molecular docking results indicated that ammonia exhibited the second least atomic contact energy (ACE) of −15.83 kcal/mol with *Paracoccus denitrificans* nitrous oxide reductase (N_2_OR) and an ACE of −15.20 kcal/mol with *Hyphomicrobium denitrificans* nitrite reductase (NIR). The inhibition of both the target enzymes (N_2_OR and NIR) supports the view of a low denitrification property and suggests the potential future applications of natural/synthetic compounds as significant nitrification inhibitors.

## 1. Introduction

Nitrogen compounds are major pollutants of wastewater, owing to their involvement in the eutrophication process and their impact on the oxygen content of receiving waters. These compounds also pose toxicity risks to aquatic species (invertebrate and vertebrate) and humans [1]. Conventional nitrification/denitrification systems have been developed and applied globally to address nitrogen elimination. Ammonia elimination is generally attained by nitrifying microbes nourished on aerated surfaces in a “biological filter” [2]. Once ammonia is removed to acceptable levels by a nitrification system, an important problem is bound to arise. The combined execution of the nitrification process, along with the reduced water exchanges, causes the gradual accumulation of nitrates in recirculating aquaculture systems [3]. Nitrate (NO_3_^−^) and nitrogen (N) are known to be toxic to fish at 181 mg/liter concentration levels [4]. The biological denitrification process involves the conversion of nitrate into elemental nitrogen (completing the nitrogen cycle) with the help of microorganisms. Therefore, this is an essential method required in the current scenario.

Denitrification is well understood as the dissimilatory conversion of nitrate or nitrite into a gaseous species after energy management. Nitrate accumulation is another main issue faced by intensive aquaculture practices such as recirculating aquaculture systems (RAS). The microbes involved in denitrification (denitrifiers) are aerobic, heterotrophic bacteria, with the potential to shift to an anaerobic respiration process due to anoxic conditions such as reducing NO_3_^−^ and NO_2_^−^ to (i) nitric oxide (NO), (ii) nitrous oxide (N_2_O), and (iii) N_2_. 

The potential of denitrifiers mainly depends upon the activities of four denitrification enzymes, namely, (a) nitrate reductase (NAR); (b) nitrite reductase (NIR); (c) nitric oxide reductase (NOR); and (d) nitrous oxide reductase (N_2_OR), which are crucial in the nitrogen cycle. These enzymes are involved in the conversion of nitrous oxide (N_2_O) into N_2_. However, some denitrifiers lack these enzymes and therefore result in N_2_O as the end product. Structurally, the enzyme is a homo-dimeric protein, where the catalytic subunit of the enzyme is encoded by the nosZ gene [5]. Medicinal plants such as *Mentha arvensis* (essential oil), *Pongamia glabra* (karanja), *Azadirachta indica* (seed oil), and *Artemisia annua* have been reported to inhibit both urea hydrolysis and nitrification [6,7,8]. Zhao and colleagues [9] studied 48 plant extracts extracted with aqueous (water) and ethanol. Among these 48 plant extracts, the aqueous extracts of *Epimeredi indica* (aerial) and *Melia azedarach* (leaf) showed good urease and nitrification inhibition (NI) activities. The *Pinus radiata* (bark) ethanolic extract has been demonstrated to reduce nitrification, microbial biomass, carbon dioxide emissions, and urease activity [10]. Similarly, the *Acacia caven* (bark) and *Azadirachta indica* (seed kernel) extracts have been reported to inhibit urease activity [11]. Few other plants have been reported to secrete nitrification inhibitors in the rhizosphere of the soil and thus inhibit the nitrification process [12]. Similarly, legume crops, such as *Arachis hypogaea* (ground nut), *Sorghum bicolor* (sorghum), and *Pennisetum glaucum* (pearl millet), have been demonstrated to possess biological nitrification inhibition (BNI) in root exudate [13]. With reference, pasture grasses, such as *Brachiaria decumbens* and *B*. *humidicola,* have been shown to possess biological nitrification inhibition (BNI) activity via arresting both the hydroxylamine oxido-reductase [HAO] and ammonia mono-oxygenase [AMO] pathways of *Nitrosomonas* [13]. The phenolic root exudates of plants have been demonstrated to inhibit the nitrification process via inhibiting nitrogen-fixing bacteria, including *Nitrosomonas europea* [13,14]. Gallocatechin (phenolic compound) has been demonstrated to inhibit nitrification in a culture of *Nitrosomonas europaea* [15]. Similarly, compounds such as brachialactone [16], 1,9-decanediol [17], methyl 3-(4-hydroxyphenyl) propionate [14], safuranetin, and sorgoleone [18] have been reported to inhibit the nitrification of *Nitrosomonas europaea*. Caffeic acid, chlorogenic acid, condensed tannins, ellagic acid, ferulic acid, gallic acid, and hydrolysable tannins have been shown to inhibit nitrification at concentrations as low as 10^−4^ to 10^−8^ M [19]. Flavonoids such as isoquercitrin, myricetin, and quercetin have been shown to inhibit ammonia oxidation via ammonium-oxidizing bacteria (AOB), namely *Nitrosomonas* [19]. Three phenolic compounds, namely ferulic acid, vanillic acid, and tannic acid, have been demonstrated to reduce N_2_O emissions [20] via protein binding and a nitrogen immobilization mechanism [21]. Adamczyk and colleagues [22] reported that larger terpenes exhibit an identical effect of reduced soil nitrogen mineralization and nitrification, as observed with mono-terpenes. Both caffeic acid and curcumin have been reported to inhibit ammonia-oxidizing archaea (AOA), especially *Nitrosomonas maritimus* [23]. Allicin (from *Allium* species) has been reported to inhibit soil urease activity [11]. Furthermore, two sulfur compounds, namely allylsulfide and allyldisulfide (from *Allium* species), have been demonstrated to inhibit bacterial ammonia mono-oxygenase [AMO] activity via an irreversible inhibition mode [19]. Ferulic acid and gallic acid have been reported to inhibit biological nitrification activity by acting as outer membrane permeabilizer agents [19]. Resveratrol has been shown to inhibit the nitrification process [24]. Gao and Zhao [25] studied the efficacy of utilizing dietary phytochemicals (such as anthocyanin, gallic acid, tannin, and tannic acid) to mitigate nitrous oxide (N_2_O) emissions. Interestingly, they showed that tannin and tannic acid as dietary supplement agents reduce the nitrous oxide (N_2_O) emissions from cattle excreta by transporting their nitrogen excretion from urine to feces. However, anthocyanin and gallic acid as dietary supplement agents reduce urine nitrous oxide (N_2_O) emissions themselves. Furthermore, the mechanisms of inhibition and the potency of these compounds can differ based on the concentration, experimental conditions, and host organism [26]. In this regard, in silico screening on the mechanisms of natural and synthetic compounds is needed to fully understand the potential of these natural compounds as inhibitors of N_2_OR and NIR. 

Thus, the aim of the present work is to carry out the in silico screening of 22 synthetic and natural compounds (ammonia, arabinoxylan, anthracene-1-carbonyl azide, 2-anthracene carboxylic acid azide, benzyl azide, 4-chloro-5-dimethylamino-2-phenyl-3-(2H)-pyridazinone, dicyandiamide, 3,5-dimethylpyrazole, 3,4-dimethylpyrazole phosphate, ethyl azide, 5-iodonaphthyl-1-azide, methylazide, 1-naphthyl azide, nicotinoylazide, 2-nitrophenyl azide, phenyl azide, procyanidin [A_1_, A_2_, B_1_, and B_2_], thiocyanate, and vitamin C) through (i) a protein network interaction analysis using a STITCH analysis, (ii) to determine their docking potential with the nitrous oxide reductase (N_2_OR) and nitrite reductase (NIR) enzymes of *Paracoccus denitrificans* PD1222 and *Hyphomicrobium denitrificans*, respectively, using PatchDock, (iii) the detection of the insecticide-likeness property using the SwissADME free online server, and (iv) predicting the bee, protozoa, and rodent toxicities using the BeeTox and pkCSM free online servers. 

## 2. Materials and Methods

### 2.1. Ligand Preparation

The chemical structures of the ligands, namely (a) Ammonia (CID 222); (b) Arabinoxylan [(CID 6438923)-]; (c) Anthracene-1-carbonyl azide [(CID 182764)-]; (d) 2-Anthracene carboxylic acid azide [(CID 102488485)-]; (e) Benzyl azide [(CID 12152)-]; (f) 4-Chloro-5-dimethylamino-2-phenyl-3-(2H)-pyridazinone [(CID 77298)-]; (g) Dicyandiamide [(CID 10005)-]; (h) 3,5-Dimethylpyrazole [(CID 6210)-]; (i) 3,4-Dimethylpyrazole phosphate [(ID 9621717)-]; (j) Ethyl azide [(CID 79118)-]; (k) 5-Iodonaphthyl-1-azide [(CID 3035415)-]; (l) Methyl azide [(CID 79079)-]; (m) 1-Naphthyl azide [(CID 123242)-]; (n) Nicotinoylazide [(CID 19914)-]; (o) 2-Nitrophenyl azide [(CID 73693)-]; (p) Phenyl azide [(CID 69319)-]; (q) Procyanidin A1 [(ID 552773)-*]; (r) Procyanidin A2 [(ID 110541)-]; (s) Procyanidin B1 [(ID 9425166)-]; (t) Procyanidin B2 [(ID 109417)-*]; (u) Thiocyanate [(CID 9322)-], and (v) Vitamin C [(CID 54670067)-], were obtained from the Chemspider and PubMed (www.pubmed.com (accessed on 26 April 2023)) databases accessed on 26 April 2023, respectively. The selected ligands were drawn in ChemBioDraw Ultra 12.0 and then a molecular mechanics (MM2) minimization of the ligands was performed using ChemBio3D Ultra 12.0 (www.cambridgesoft.com). Thus, these energy-minimized structures (ligands) were further utilized for the PatchDock study. 

### 2.2. Protein Network Interaction Analysis

“The search tool for interacting chemicals” (STITCH) free-online server provides detailed information about the following; (a) metabolic pathway interactions; (b) crystal structure information; (c) binding potential; and (d) target-drug correlations [27]. In the present study, the STITCH online tool [28] was used for identifying the interactions between the twenty-two selected ligands and the proteins of the target organisms (*Paracoccus denitrificans* and *Hyphomicrobium denitrificans*).

### 2.3. Prediction of Insecticide-Likeness Property

In the agro-chemical discovery and development, Lipinski’s rule of five (Ro5) filter was utilized to assess agro-chemical natures such as herbicides, insecticides, and pesticides. In this regard, Tice [29] adopted Lipinski’s rule of five (Ro5) molecular descriptors (molecular weight; lipophilicity/hydrophobicity; number of hydrogen bond donors and acceptors; and number of rotatable bonds) as significant criteria for determining herbicidal, insecticidal, and pesticidal properties [30]. Thus, in the current study, the SwissADME free online server was used to predict the insecticide-likeness property of the selected 22 (synthetic and natural compounds) ligands [31].

### 2.4. Prediction of Toxicity

BeeTox is an artificial intelligence (AI)-based free online server used to predict the acute toxicity of chemicals/ligands to honey bees [32]. In the present study, the toxicity of the chosen ligands towards protozoa, bacteria (*Tetrahymena pyriformis*), and rodents (rat) was assessed using the BeeTox and pkCSM free online servers [31,32].

### 2.5. Target Protein Identification and Preparation

The three-dimensional (3D) structures of the *Paracoccus denitrificans* nitrous oxide reductase (PDB ID: 1FWX with a resolution of 1.6 A°) and *Hyphomicrobium denitrificans* nitrite reductase (PDB ID: 2DV6 with a resolution of 2.2 A°) were downloaded from the Research Collaborator for Structural Bioinformatics (RCSB) Protein data bank (www.rcsb.org). The “A” chains of both the selected proteins were pre-processed separately by deleting the other chains and ligands (except copper), as well as the crystallographically observed water molecules (water without hydrogen bonds). Both the proteins were prepared using the UCSF Chimera software (www.cgi.ucsf.edu/chimera) and the resultant proteins were further utilized for the PatchDock study.

### 2.6. PatchDock Study

The docking studies were carried out using the PatchDock free web-based server (http://bioinfo3d.cs.tau.ac.il/PatchDock). It adopts a geometry-based molecular docking algorithm method and is also utilized to recognize the binding scores, binding residues, and atomic contact energy of chosen ligands [31]. Generally, the docking results are obtained through the user’s email address. We used a uniform resource locator (URL), which would provide the top 20 solutions in a table form via a user email. From these, the top one (the docked protein–ligand complex), which denoted the best solution, was selected and downloaded in the program database (PDB) file format. Finally, the binding site analyses were carried out using the PyMOL software (www.pymol.org).

## 3. Results and Discussion

Soil microbes play a vital role in nitrogen cycling, especially in terrestrial ecosystems, and they are also involved in significant transformation steps, such as nitrogen fixation, nitrification, and denitrification [26]. About 80% of the global emissions of nitrous oxide (N_2_O), which are 300 times higher than those of carbon dioxide (CO_2_) emissions, may be due to the over-production and application of nitrogen fertilizers in the agriculture sector. Annual nitrogen fertilizer application will reach around 300 teragrams (Tg) by the year 2050, which will result in 7.5 Tg of nitrous oxide (N_2_O) emissions [33].

Nitrification inhibitors (NI) are known to alleviate the nitrate-leaching process and have also been demonstrated to decrease the nitrous oxide (N_2_O) emission rate, especially after the use of nitrogen fertilizers. In recent years, 3,4-dimethyl pyrazole phosphate (DMPP) has gained much attention among scientists and exhibited an advantage over dicyandiamide (DCD), another nitrification inhibitor [34]. Similarly, it has been reported that *Fallopia* species inhibit the denitrification process by releasing procyanidins via a process called BDI (“biological denitrification inhibition”) [35,36].

Nitrification inhibitors using commercial compounds have minimal accessibility and an unfavorable impact on the ecosystem [37]. Herbal derivatives such as essential oils and oil cakes have been employed to block the nitrification process in soil in an ecologically safer direction [38]. A thorough literature search shows that plants and their bioactive products are capable of inhibiting the nitrification process in different soils. Sahrawat and Mukherjee [39] reported that *Pongamia glabra* (Indian Beech tree) seed extracts possess nitrification-inhibiting (NI) activity in different soil samples. Similarly, oil cakes derived from *Citrullus colocynthis* (bitter cucumber) have been reported to possess significant nitrification inhibitor activity compared to that of urea, with a 67% efficiency under both laboratory and greenhouse assays [40]. *Azadirachta indica* (Neem tree) seeds have been reported to exhibit the deceleration of nitrification of urea (nitrogenous fertilizer), specifically in soil with a pH of more than 6.0 [37]. Essential oils derived from *Madhuca indica* (Indian butter tree) and *Onosma hispidum* (Ratanjot) have been reported as potent nitrification inhibitors (NI) on diverse soil samples [41]. Prasad and Power [42] showed that the waste extracts of *Camelia sinensis* (Green tea), along with their bioactive compounds, including polyphenols, displayed a significant inhibition of soil nitrification. The flower dust derived from *Chrysanthenum cinerariefolium* (Pyrethrum daisy) has been reported as a potent nitrification inhibitor (NI) and to improve N use efficiency two-fold compared to prilled urea [43]. *Artemisia annua* (Sweet sagewort) leaf extracts containing the major metabolite artemisinin (a sesquiterpene) have exhibited significant nitrification inhibition (NI) actions on different soil samples under in vitro conditions [44]. Three native herbaceous perennial plants of *Ethiopia artemis* afra (Mugwort), *Echinops* spp. (Pale globe-thistle), and *Eugenia caryophyllata* (Clove) have demonstrated significant nitrification-inhibiting (NI) actions [45]. Moreover, the essential oils derived from *Mentha spicata* (Spearmint) have exhibited a deceleration of nitrification in the soil as compared to urea. The average NO_3_-N formation was minimal in the urea treatment compared to that of essential oils [46]. *Brachiaria humidicola* (Koronivia grass) root tissue extracts have been reported as nitrification inhibitors [47]. *Linum usitatissimum* (Linseed) essential oil has been found to exhibit nitrification-inhibiting (NI) activity [37]. *Sorghum bicolor* (Indian millet) root extracts showed significant nitrification inhibition (NI) under in vitro conditions in different soils [48]. Different crude extracts of *Cinnamomum verum* (Cinnamon), *Madhuca longifolia* (Madhuka), *Lantana camara* (Lantana), *Myristica fragrans* (nutmeg), and *Piper nigrum* (Black pepper) have shown the deceleration of nitrification with less non-target impacts on different soils [41]. Similarly, synthetic chemicals such as acetylene, azide, CO, and cyanide also act as NO inhibitors of *Paracoccus denitrificans*, a major nitrate-reducing microbe [49]. The above survey illustrates well that natural and chemical compounds have a greater potential to inhibit the nitrification process.

To improve the yield of crops, farmers fortify soils with different nutrients, including nitrogen fertilizers. Though, whenever applied, nitrogen fertilizers are not entirely allotted to plants. These efforts lead to the loss of diverse mechanisms such as the transformation of NO_3_^−^ into N_2_O and N_2_ through the denitrification process [50]. Earlier, Galland et al. [51] reported that the denitrification inhibition process enhances the plant growth and nutrition index of *Apium graveolens* L. (Celery) for a longer period. Procyanidins are polyphenolic compounds composed of condensed flavan-3-ol moieties. Procyanidins vary depending upon their monomers of (−)-epicatechin/(+)-catechin, forming oligomeric/polymeric structures, which are commonly found in apples, grapes, and sweet violets, etc. [52]. In the present study, 22 selected (synthetic and natural compounds) ligands (as shown in Table 1) were evaluated for their docking behavior with *Paracoccus denitrificans* nitrous oxide reductase (N_2_OR) and *Hyphomicrobium denitrificans* nitrite reductase (NIR) using PatchDock.

Table 2 shows the insecticide-likeness properties of the selected 22 (synthetic and natural compounds) ligands, where nine ligands, namely anthracene-1-carbonyl azide, 2-anthracene carboxylic acid azide, benzyl azide, 4-chloro-5-dimethylamino-2-phenyl-3-(2H)-pyridazinone, 3,5-dimethylpyrazole, 5-iodonaphthyl-1-azide, 1-naphthyl azide, 2-nitrophenyl azide, and phenyl azide, comply well with the TICE rule.

Similarly, Table 3 shows the toxicity analysis of the selected 22 (synthetic and natural compounds) ligands, where none of the ligands have any toxicity towards honey bees.

**Figure 1:**
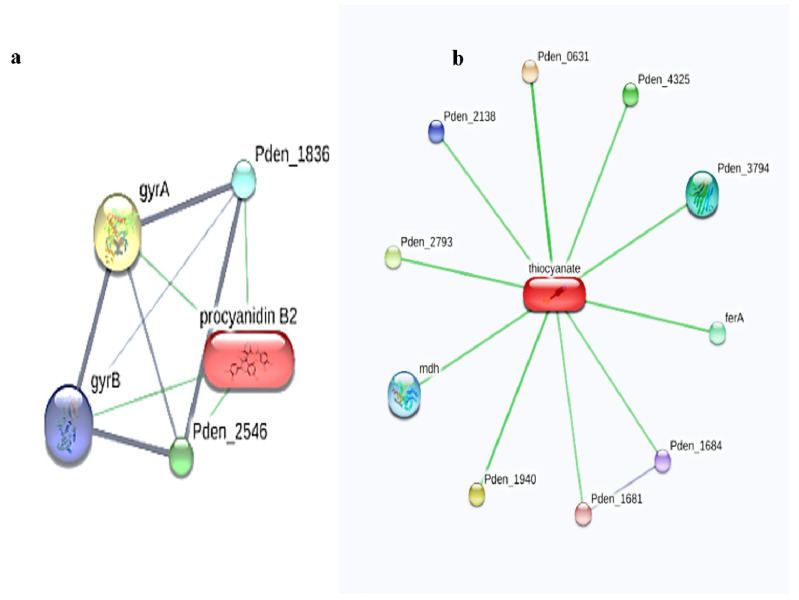
Ligand–protein interactions of (**a**) procyanidin B2 and (**b**) thiocyanate interactions with the *Paracoccus denitrificans* PD1222 proteins using STITCH web server.

**Figure 2:**
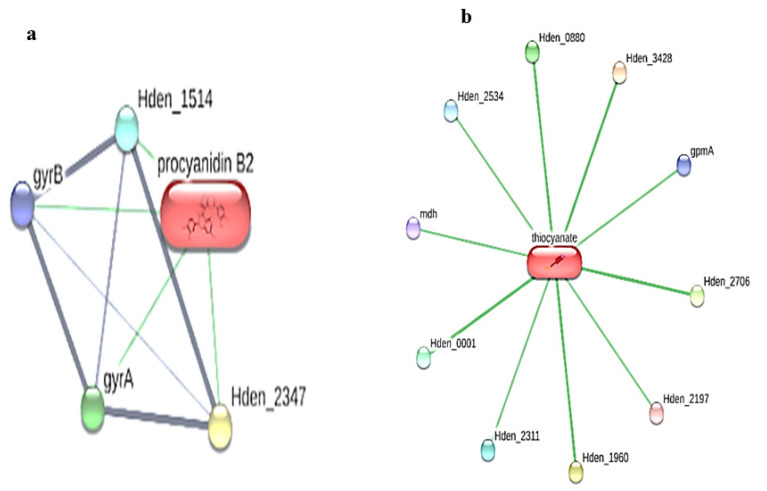
Ligand–protein interaction of (**a**) procyanidin B2 and (**b**) thiocyanate interactions with the *Hyphomicrobium denitrificans* proteins using STITCH web server.

Interestingly, in the present study, the STITCH analysis revealed that two ligands, namely procyanidin B2 and thiocyanate, exhibited interactions with both the *Paracoccus denitrificans* PD1222 (Figure 1) and *Hyphomicrobium denitrificans* proteins (Figure 2), respectively.

The nitrous oxide reductases’ (N_2_OR) enzymes have been reported from the following microorganisms, such as (i) *Achromobacter cycloclastes*, (ii) *Alcaligenes faecalis* IAM 1015, (iii) *Alcaligenes* sp. NCIB 11015, (iv) *Flexibacter canadensis*, (v) *Paracoccus denitrificans*, (vi) *Pseudomonas aeruginosa*-P2, (vii) *Pseudomonas stutzeri*, (viii) *Rhodobacter capsulatus*, (ix) *Rhodobacter sphaeroides* f.sp. *denitrificans*, (x) *Thiosphaera pantotropha,* and (xi) *Wolinella succinogenes* [53]. Among these, *Paracoccus denitrificans* nitrous oxide reductase enzymes have been exclusively reported by researchers [49]. The docking (in silico) studies and binding site analyses demonstrated that arabinoxylan has the maximum atomic contact energy (ACE) of −188.05 (kcal/mol), while procyanidin B1 has the lowest ACE of −1.85 (kcal/mol) with the *Paracoccus denitrificans* nitrous oxide reductase (as shown in Table 4).

Similarly, we observed the atomic contact energy (ACE) in the following order: Arabinoxylan; < Nicotinoylazide; < 2-Nitrophenyl azide; < Benzyl azide; < Phenyl azide; < Thiocyanate; < Procyanidin A2; < Procyanidin A1; < Procyanidin B2; < 3,5-Dimethylpyrazole; < Dicyandiamide; < 5-Iodonaphthyl-1-azide; < Anthracene-1-carbonyl azide; < 1-Naphthyl azide; < 4-Chloro-5-dimethylamino-2-phenyl-3-(2H)-pyridazinone; < Vitamin C; < Ethyl azide; < 3, 4-Dimethylpyrazole phosphate; < 2-Anthracene carboxylic acid azide; < Methyl azide; < Ammonia; < Procyanidin B1. This current finding is in good agreement with the earlier study, where azide and cyanide have been reported to inhibit *Paracoccus denitrificans* nitrous oxide reductase (N_2_OR) activity [54]. Interestingly, procyanidin B1 has been demonstrated to interact with the His73 amino acid (A.A) residue of *Paracoccus denitrificans* nitrous oxide reductase (N_2_OR), as shown in Table 4. Procyanidins have been reported to inhibit the denitrification in *Pseudomonas brassicacearum* [55]. Thiocyanate (SCN^−^) has been demonstrated to inhibit the reduction of nitrous oxide (N_2_O) produced for nitrate–nitrogen in growing cells of *Paracoccus denitrificans* [56]. *Azadirachta indica* (Neem) phytochemicals, namely azadirachtin, diepoxy azadiradione, dyhidrogedunin, gallic acid, gedunin, nimbin, nimbidin, nimbic acid, nimbidinin, and nimbinin, have been reported to dock with the nitric oxide reductase of *Pseudomonas aeruginosa* [57]. Among these phytochemicals, diepoxy azadiradione showed the least binding energy (−8.7 kcal/mol) with that of the *Pseudomonas aeruginosa* nitric oxide reductase (N_2_OR). To the best of our knowledge, this is the first ever report for these 22 compounds.

Nitrite reductase (NIR) has been reported from the following microorganisms: (i) *Achromobacter cycloclastes*, (ii) *Alcaligenes faecalis*, (iii) *Alcaligenes sphaeroides*, (iv) *Bradyrhizobium japonicum*, (v) *Desulfovibrio desulfuricans*, (vi) *Escherichia coli*, (vii) *Fusarium oxysporum*, (viii) *Hyphomicrobium nitrativorans*, (ix) *Hyphomicrobium denitrificans*, (x) *Hyphomicrobium zavarzinii*, (xi) *Nitrosomonas europaea*, (xii) *Paracoccus denitrificans*, (xiii) *Pseudomonas aeruginosa*, (xiv) *Pseudomonas aureofaciens*, (xv) *Rhodopseudomonas sphaeroides*, (xvi) *Thiobacillus denitrificans*, (xvii) *Vibrio alginolyticus*, (xviii) *Vibrio fischeri*, and (xix) *Wolinella succinogenes* [56,57,58,59,60]. Among these, *Hyphomicrobium* species are facultative methylotrophs, commonly found in water and soil and also isolated from sewage treatment plants [59].

The docking studies showed that procyanidin B1 has the maximum ACE of −250.92 (kcal/mol), whereas ammonia has the lowest ACE of −15.20 (kcal/mol) with the *Hyphomicrobium denitrificans* nitrite reductase (Table 5).

From the studies, we observed the atomic contact energy (ACE) in the following order: Procyanidin B1; < Procyanidin B2; < Procyanidin A1; < Procyanidin A2; < 4-Chloro-5-dimethylamino-2-phenyl-3-(2H)-pyridazinone; < 5-Iodonaphthyl-1-azide; < Arabinoxylan < 2-Nitrophenyl azide; < 1-Naphthyl azide; < Benzyl azide; < Nicotinoylazide; < Phenyl azide; < 2-Anthracene carboxylic acid azide; < 3,4-Dimethylpyrazole phosphate; < Vitamin C; < 3,5-Dimethylpyrazole; < Anthracene-1-carbonyl azide; < Thiocyanate; < Methyl azide; < Ethyl azide; < Dicyandiamide; < Ammonia. This current finding is concurrent with the previous study, where 3,4-Dimethylpyrazole phosphate (DMPP) was reported to inhibit bacterial nitrite reductase (NIR) activity [61]. In the present study, six ligands, namely 3,4-dimethyl pyrazole phosphate, 5-iodonaphthyl-1-azide, methyl azide, nicotinoylazide, 2-nitrophenyl azide, and thiocyanate, were found to interact with the Leu421 amino acid (A.A) residue of *Hyphomicrobium denitrificans* nitrite reductase (NIR), as shown in Table 5. Ten phytochemicals of neem (*Azadirachta indica*), such as azadirachtin, diepoxy azadiradione, dyhidrogedunin, gallic acid, gedunin, nimbin, nimbidin, nimbic acid, nimbidinin, and nimbinin were demonstrated to bind with that of the *Paracoccus pantotrophus* cytochrome CD1 nitrite reductase [57]. Among these phytochemicals, both the nimbidin and nimbidinin showed the least binding energy (−8.3 kcal/mol) with that of the *Paracoccus pantotrophus* cytochrome CD1 nitrite reductase (NIR). However, no docking reports are available for these 22 ligands to date.

## 4. Conclusions

In conclusion, all the selected ligands displayed docking capability with both of the targeted enzymes (N_2_OR and NIR). Interestingly among the 22 ligands, ammonia exhibited the second-lowest atomic contact energy with the nitrite reductases (NIR) of *Paracoccus denitrificans* and *Hyphomicrobium denitrificans*. The inhibition of both enzymes (N_2_OR and NIR) illustrates the nitrification inhibition potential of these 22 compounds and paves an enhanced view of the future applications of natural/synthetic compounds as significant nitrification inhibitors. Despite this, detailed in vitro screening on the mode of action of the selected compounds responsible for the denitrification process, along with microbial assays of urease activity, are required.

## Figures and Tables

**Table 1:** Two-dimensional (2D) structures of 22 selected (synthetic and natural compounds) ligands.

Ligand Name	Structure
Ammonia	NH_3_
Arabinoxylan	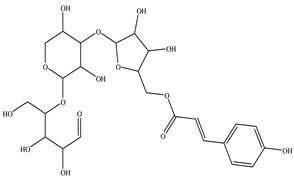
Anthracene−1−carbonyl azide	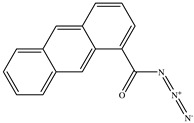
2−Anthracene carboxylic acid azide	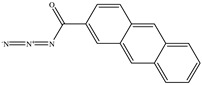
Benzyl azide	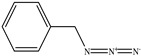
4−Chloro−5−dimethylamino−2−phenyl−3− (2H)−pyridazinone	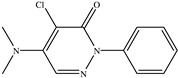
Dicyandiamide	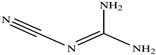
3,5−Dimethylpyrazole	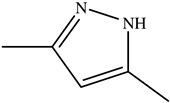
3, 4−Dimethylpyrazole phosphate	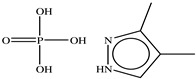
Ethyl azide	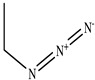
5−Iodonaphthyl−1−azide	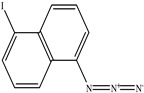
Methyl azide	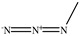
1−Naphthyl azide	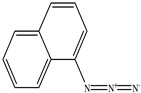
Nicotinoylazide	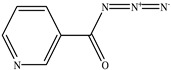
2−Nitrophenyl azide	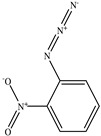
Phenyl azide	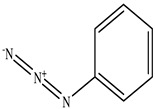
Procyanidin A1	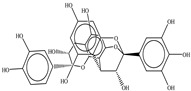
Procyanidin A2	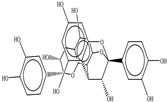
Procyanidin B1	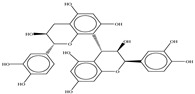
Procyanidin B2	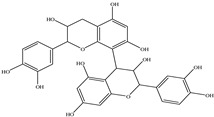
Thiocyanate	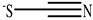
Vitamin C	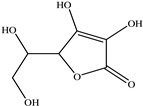

**Table 2:** Prediction of insecticide-likeness of selected 22 (synthetic and natural) compounds/ligands using SwissADME online tool.

Ligand	MW^1^	MLogP^2^	HBD^3^	HBA^4^	RB^5^	TICE Rule Violation (for Insecticide Property)
Ammonia	17.031	ND^7^	1	1	0	ND^7^
Arabinoxylan	560.50	−4.03	8	15	13	Yes
Anthracene-1-carbonyl azide	247.75	3.26	0	4	2	No
2-Anthracene carboxylic acid azide	247.25	3.26	0	4	2	No
Benzyl azide	133.15	1.18	0	3	2	No
4-Chloro-5-dimethylamino-2-phenyl-3-(2H)-pyridazinone	249.70	2.37	0	2	2	No
Dicyandiamide	84.08	−1.51	2	2	0	Yes
3,5-Dimethylpyrazole	96.13	0.35	1	1	0	No
3,4-Dimethylpyrazole phosphate	194.13	−1.50	4	5	0	Yes
Ethyl azide	71.08	−0.66	0	3	1	Yes
5-Iodonaphthyl-1-azide	295.08	3.04	0	3	1	No
Methyl azide	57.05	−1.30	0	3	0	Yes
1-Naphthyl azide	169.18	2.20	0	3	1	No
Nicotinoylazide	148.12	−0.25	0	5	2	Yes
2-Nitrophenyl azide	164.12	0.88	0	5	2	No
Phenyl azide	119.12	1.09	0	3	1	No
Procyanidin A1	592.50	−0.34	10	13	2	Yes
Procyanidin A2	576.50	0.14	9	12	3	Yes
Procyanidin B1	578.52	−0.26	10	12	3	Yes
Procyanidin B2	578.52	−0.26	10	12	3	Yes
Thiocyanate	58.08	−1.01	0	1	0	Yes
Vitamin C	176.12	−2.60	4	6	2	Yes

Note: MW^1^—Molecular weight; LogP^2^—Lipophilicity/hydrophobicity; HBD^3^—Number of hydrogen bond donors; HBA^4^—Number of hydrogen bond acceptors, RB^5^—Number of rotatable bonds, and ND^7^—Not determined.

**Table 3:** Acute toxicity prediction of 22 selected (synthetic and natural) compounds/ligands using Bee-Tox (LabMol) and predicting small-molecule pharmacokinetic and toxicity properties (pkCSM) online tool.

Ligand Name	Toxicity		
	Bee	Protozoa *	Fish **
Ammonia	ND	−2.328	2.911
Arabinoxylan	Non-toxic	0.285	8.481
Anthracene-1-carbonyl azide	Non-toxic	0.327	−0.326
2-Anthracene carboxylic acid azide	Non-toxic	0.336	−0.326
Benzyl azide	Non-toxic	0.233	1.445
4-Chloro-5-dimethylamino-2-phenyl-3-(2H)-pyridazinone	Non-toxic	0.763	1.09
Dicyandiamide	Non-toxic	−0.162	3.549
3,5-Dimethylpyrazole	Non-toxic	−0.477	2.706
3,4-Dimethylpyrazole phosphate	Non-toxic	0.126	3.096
Ethyl azide	Non-toxic	−0.851	2.349
5-Iodonaphthyl-1-azide	Non-toxic	0.506	−0.67
Methyl azide	Non-toxic	−1.056	2.514
1-Naphthyl azide	Non-toxic	0.399	0.31
Nicotinoylazide	Non-toxic	0.074	1.924
2-Nitrophenyl azide	Non-toxic	0.593	0.971
Phenyl azide	Non-toxic	0.084	1.48
Procyanidin A1	Non-toxic	0.285	8.966
Procyanidin A2	Non-toxic	0.285	7.542
Procyanidin B1	Non-toxic	0.285	8.151
Procyanidin B2	Non-toxic	0.285	8.704
Thiocyanate	Non-toxic	−0.823	2.537
Vitamin C	Non-toxic	0.285	4.386

Note: ND—Not determined, Protozoa * (*Tetrahymena pyriformis*) toxicity expressed in Log microgram/L and Fish ** (Minnow toxicity) expressed as in Log mM.

**Table 4:** Docking and interaction site analysis of 22 selected (synthetic and natural) compounds/ligands with *Paracoccus denitrificans* nitrous oxide reductases (N_2_OR) using the PatchDock online server.

Lgand	−ACE^▪^ (kcal/mol)	Interaction of Amino Acid (A.A) Residue	Bond Distance (in A°)
Ammonia	15.83	Phe498	2.52
Arabinoxylan	188.05	Arg45	2.70
		Lys114	2.28
		Thr525	3.18
		Glu538	2.83 & 3.37
		Trp563	2.18
Anthracene-1-carbonyl azide	61.89	Ser72	3.44
2-Anthracene carboxylic acid azide	47.71	Asn102	2.73
		Thr103	3.34
		Glu120	3.07
Benzyl azide	120.82	No interactions	-
4-Chloro-5-dimethylamino-2-phenyl-3-(2H)-pyridazinone	58.25	Glu120	3.12
Dicyandiamide	75.65	No interactions	-
3,5-Dimethylpyrazole	78.91	No interactions	-
3,4-Dimethylpyrazole phosphate	48.32	Thr55	2.67 & 2.76
		Thr60	3.26
		Glu538	3.36
Ethyl azide	56.82	No interactions	-
5-Iodonaphthyl-1-azide	63.94	Ser72	2.81
		Glu120	2.02
Methyl azide	28.18	Asn227	3.14
		Asp300	3.12 & 3.40
		Tyr304	2.88
1-Naphthyl azide	60.80	Leu151	3.60
Nicotinoyl azide	130.54	Thr544	2.82
2-Nitrophenyl azide	125.37	Thr55	2.68
		Thr60	3.07 & 3.08
		Thr544	3.07
Phenyl azide	111.39	No interactions	-
Procyanidin A1	95.81	Ser72	3.38
		Thr103	2.64
		Lys125	3.34
		Leu151	2.26
		Asn153	2.78 & 3.58
		Thr862	3.39
Procyanidin A2	101.08	Ser72	3.28
		Asn123	2.59
		Ala124	3.40
		Gly126	3.16
		Asn244	3.09
Procyanidin B1	1.85	Ser72	3.55
		His73	2.86
		Asn123	3.01
		Asn244	3.38
Procyanidin B2	83.73	Thr103	3.28
		Ile121	2.82
		Arg861	2.50
		Thr862	2.50 & 3.15
Thiocyanate	105.42	Arg254	3.54
Vitamin C	57.47	Thr55	2.48
		Thr60	3.27 & 3.49

Note: −ACE^▪^—Atomic contact energy.

**Table 5:** Docking and interaction site analysis of 22 selected (synthetic and natural) compounds/ligands with *Paracoccus denitrificans* nitrite reductase (NIR) using the PatchDock online server.

Ligand	−ACE^▪^ (kcal/mol)	Interaction of Amino Acid (A.A) Residue	Bond Distance (in A°)
Ammonia	15.20	No interactions	-
Arabinoxylan	169.95	Thr336	1.96 & 3.54
Anthracene-1-carbonyl azide	104.61	Thr336	2.58
2-Anthracene carboxylic acid azide	126.37	Ala136	3.14
Benzyl azide	151.70	No interactions	-
4-Chloro-5-dimethylamino-2-phenyl-3-(2H)-pyridazinone	203.06	Pro26	2.38
Dicyandiamide	50.91	Asp179	3.14
3,5-Dimethylpyrazole	107.94	No interactions	-
3,4-Dimethylpyrazole phosphate	118.81	Leu421	2.78
Ethyl azide	57.42	Asp178	2.63
		Asp179	2.95 & 3.43
5-Iodonaphthyl-1-azide	180.56	Leu421	3.30
Methyl azide	63.85	Leu421	2.85, 3.03 & 3.16
1-Naphthyl azide	153.91	No interactions	-
Nicotinoyl azide	144.25	Leu421	3.13
2-Nitrophenyl azide	156.39	Leu421	2.95 & 3.11
Phenyl azide	131.36	No interactions	-
Procyanidin A1	211.30	Thr336	2.57
		Arg337	3.55
Procyanidin A2	207.69	Thr336	2.39
Procyanidin B1	250.92	Asp178	2.92
Procyanidin B2	240.34	Pro26	2.41
		Val28	2.69
Thiocyanate	76.65	Leu421	3.55
Vitamin C	112.51	No interactions	-

Note: −ACE^▪^—Atomic contact energy.

## Data Availability

All the data details are available with the corresponding authors, if any require the same can contact him for the same.
